# The complete chloroplast genome and phylogenetic analysis of *Salvia karwinskii* (Lamiaceae)

**DOI:** 10.1080/23802359.2022.2101398

**Published:** 2022-07-28

**Authors:** Guiping Zhao, Yifei Pei, Dade Yu, Furong Xu, Xiwen Li

**Affiliations:** a College of Traditional Chinese Medicine, Yunnan University of Chinese Medicine, Kunming, China; b Institute of Chinese Materia Medica, China Academy of Chinese Medical Sciences, Beijing, China

**Keywords:** *Salvia karwinskii*, complete chloroplast genome, phylogenetic analysis

## Abstract

*Salvia karwinskii* Benth. 1835 is a perennial herb in the Lamiaceae family native in Mexico and Central America. The complete chloroplast (cp) genome of *S. karwinskii* was sequenced using the Illumina platform and assembled for the first time. The complete plastid genome of *S. karwinskii* was 150,907 bp in length including a large single-copy (LSC) region of 82,205 bp, a small single-copy (SSC) region of 17,538 bp, and a pair of inverted repeat (IR) regions of 25,582 bp. The total GC content of this genome was 38.05%, and that of LSC, SSC, and IR regions was 36.22%, 31.77%, and 43.14%, respectively. The cp genome contained 114 unique genes, including 80 protein-coding genes, 30 tRNA genes, and four rRNA genes. The maximum-likelihood phylogenetic tree was constructed with 38 complete cp genomes, supporting a close relationship between *S. karwinskii* and a 10 species lineage, all of which belong to the subg. *Calosphace* of *Salvia*. The cp genome of *S. karwinskii* provides a foundation for further studies on genetic diversity and improving the classification system of *Salvia*.

*Salvia* is the largest genus in the family Lamiaceae, containing appropriately 1000 species of shrubs, herbaceous perennials and annuals (Li et al. 2013; Cui et al. 2020). Many species of *Salvia* are popular garden plants because they typically bloom for a long period of time and grow well even in harsh conditions (Hu et al. 2020; Zhou et al. 2021). *Salvia karwinskii* Benth. 1835 is a tall and evergreen perennial shrub belonging to the genus *Salvia*. It is widely distributed throughout Mexico and Central America, including Guatemala, Honduras, El Salvador, and Nicaragua. *S. karwinskii* produces watermelon-pink blooms that form lower-loose and upper-dense racemes. It has great ornamental value, with a long blooming time from May to December every year. However, its research in genetics and evolution is extremely rare. In this study, we sequenced the chloroplast (cp) genome of *S. karwinskii* and examined its phylogenetic position within the genus *Salvia*. It is expected to lay the foundation for further breeding studies of *S. karwinskii.*

Fresh leaves of *S. karwinskii* were collected from Guatemala Botanical Garden, Guatemala (90°30′ N, 44°02′ W), and identified by Xiwen Li. The specimen was deposited at herbarium of the Institute of Chinese Materia Medica, China Academy of Chinese Medicinal Sciences, Beijing, China (http://www.icmm.ac.cn/, Xiwen Li, xwli@icmm.ac.cn) under the voucher number SZ20190920. The total genomic DNA was extracted by the modified cetyltrimethylammonium bromide (CTAB) method (Doyle and Doyle 1987). The total DNA was used to generate a library with an average insertion size of 350 bp. The cp genome sequencing was performed on the Illumina Hiseq 1500 platform (Illumina Inc., San Diego, CA) with the paired-end 150 bp strategy. The complete cp genome of *S. miltiorrhiza* (NC020431) was used as the reference genome for extracting cp genome reads (Qian et al. 2013). It was assembled by SOAPdenovo (version 2.04) (Luo et al. 2012). The complete cp genome of *S. karwinskii* (accession number MT156372) was submitted to GenBank after being annotated by Plann (Huang and Cronk 2015).

The complete cp genome of *S. karwinskii* was 150,907 bp in length including a large single-copy (LSC) region of 82,205 bp, a small single-copy (SSC) region of 17,538 bp, and a pair of inverted repeat (IR) regions of 25,582 bp. The total GC content of this cp genome was 38.05%, while 36.22%, 31.77%, and 43.14% in the LSC, SSC, and IR regions, respectively. Besides, 114 unique genes were obtained in the cp genome, including 80 protein-coding genes, 30 tRNA, genes and four rRNA genes.

A total of 34 cp genomes of *Salvia* species were downloaded from GenBank. The complete cp genomes including both IR regions of these 34 Salvia species and *Salvia karwinskii* were used for phylogenetic analysis, together with *Mentha longifolia*, *M. canadensis* as well as *Glechoma longituba* as outgroups. After alignment with MAFFT (version 7.310) (Rozewicki et al. 2019), the maximum-likelihood (ML) tree was conducted using RAxML (version 8.2.12) (Stamatakis 2014) with the GTRGAMMA model and 1000 bootstrap replicates. It was clear from the phylogenetic tree that the main nodes were supported with high bootstrap values (Figure 1). The topology based on entire cp genomes showed that *Salvia* species were monophyletic and clustered into three clades. These clades just corresponded to the three distribution centers of *Salvia*, which were Central and South America, Central Asia-Mediterranean and East Asia (Walker et al. 2004). The ML tree also showed that *S. karwinskii* was embedded within the ‘Central and South America’ clade. The ‘Central and South America’ clade could be divided into two subclades: *S. madrensis* and a small lineage including *S. karwinskii* and 10 other subg. *Calosphace* species. Our phylogenetic analysis revealed a close relationship between *S. karwinskii* and a 10 species lineage, all of which belong to the subg. *Calosphace* of *Salvia*. In conclusion, this newly assembled cp genome could serve as a foundation for further better cultivation and utilization of *S. karwinskii*.

**Figure 1.**
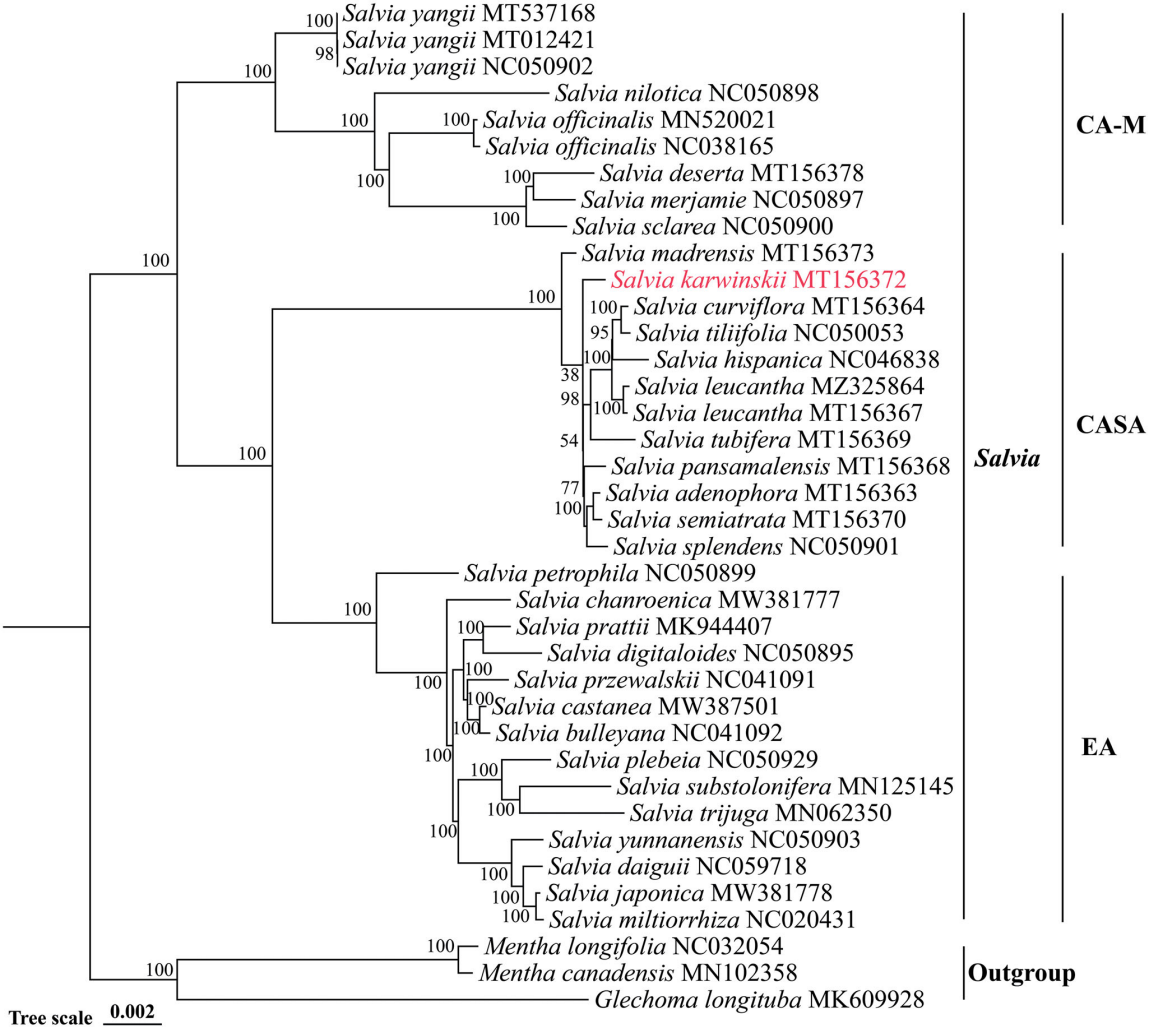
The maximum-likelihood (ML) tree was constructed based on complete chloroplast genome sequences of 35 *Salvia* species. *Mentha canadensis*, *M. longifolia*, and *Glechoma longituba* were used as outgroups. Bootstrap values with 1000 replicates were shown under each branch. CA-M: Central Asia-Mediterranean; CASA: Central and South America; EA: East Asia.

## Data Availability

The chloroplast genome sequence that supports the findings of this study are openly available in GenBank of National Center for Biotechnology Information (NCBI, https://www.ncbi.nlm.nih.gov) under the accession number MT156372. The associated BioProject, Bio-Sample, and SRA numbers are PRJNA831670, SAMN27760424, and SRR18909709, respectively.
